# Supplementary material for: Phloem-Mobile MYB44 Negatively Regulates Expression of PHOSPHATE TRANSPORTER 1 in Arabidopsis Roots
Source: Plants (Basel). 2023 Oct 19;12(20):3617. doi: 10.3390/plants12203617 (PMC10610484; doi:10.3390/plants12203617)
Supplement: Supplementary file 1 [file plants-12-03617-s001.zip › plants-2581932-supplementary.pdf]

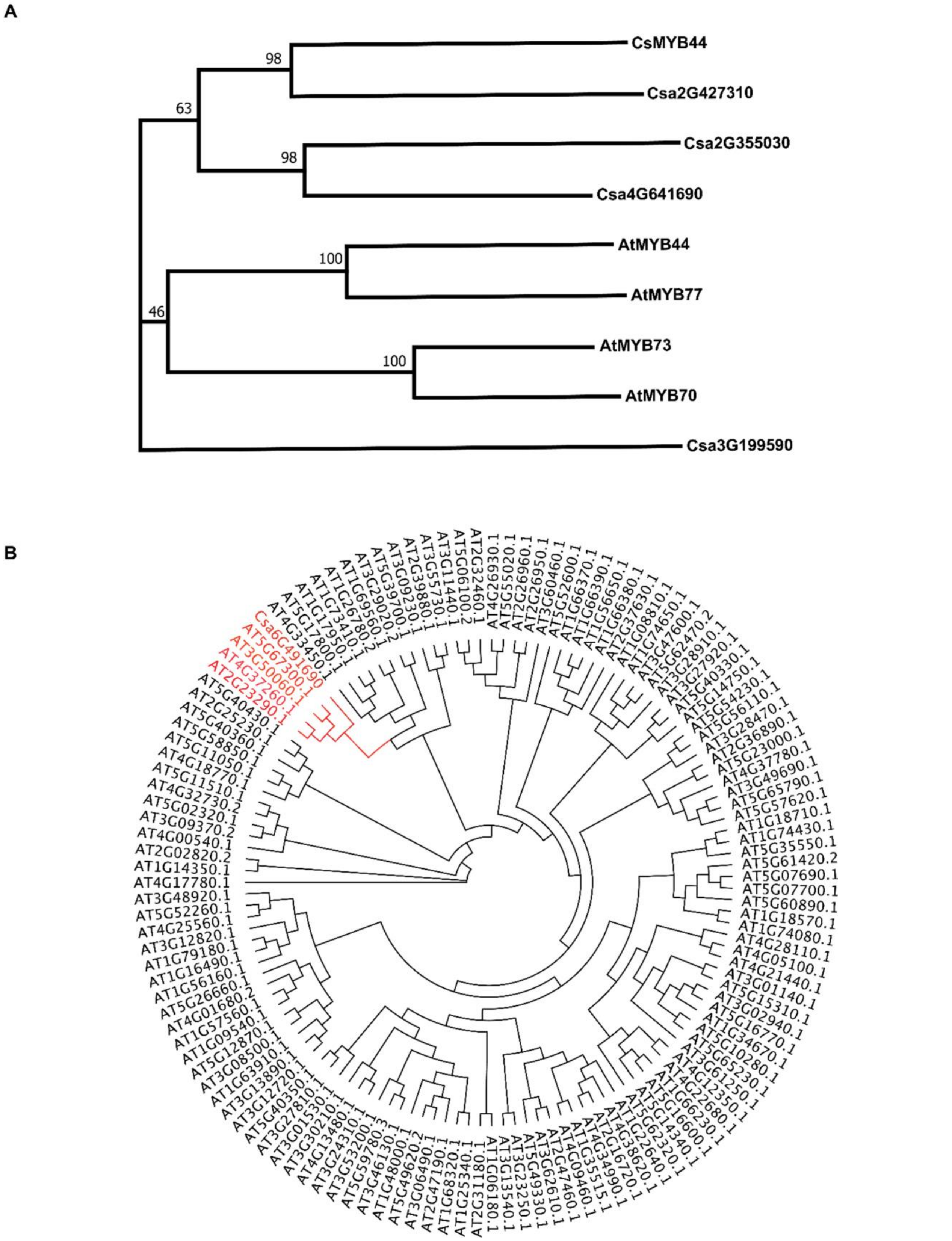

Figure S1. Phylogenetic analysis of the *A. thaliana* MYB family.

(A) The phylogenetic relationship of the MYB family in AtMYB44 and CsMYB44 clade. The numbers beside the nodes indicate bootstrap support values.

(B) The phylogenetic tree showed the relationship between the Arabidopsis MYB family and the cucumber MYB44, identified as potential phloem mobile mRNA in the previous study [29]. The clade of the MYB family, which showed the closest similarity with the cucumber MYB44, is highlighted in red.

Protein sequences of the Arabidopsis and cucumber MYB family, downloaded from The Arabidopsis Information Resource (TAIR) database and Cucurbit Genome database (CuGenDB), were aligned by using Clustal Omega (https://www.ebi.ac.uk/Tools/msa/clustalo/). The phylogenetic tree was generated with the neighbor-joining (NJ) method in Geneious software and MEGA11.

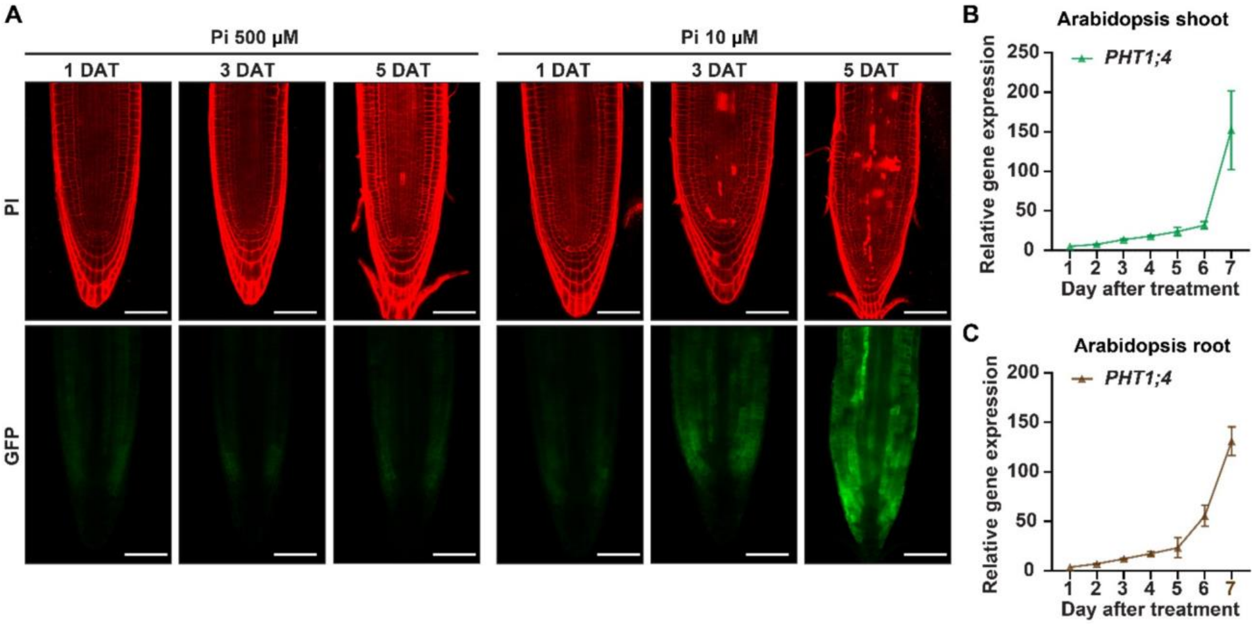

Figure S2. AtMYB44 expression was increased in response to Pi-starvation stress.
(A) *AtMYB44-GFP* expression was increased in the primary root tip under Pi-starvation (10 μM) conditions. Representative confocal images showed the GFP signal in the primary root tip after Pi-sufficient (500 μM) (K) and starvation treatment (10 μM) (L) for 1, 3 and 5 days. Arabidopsis seedling roots were stained with propidium iodide (PI, red). Epi, epidermis; Cor, cortex layer; End, endodermis. Bar = 50 μm.
(B) and (C) The expression level of *PHT1;4* in shoots (B) and roots (C) under Pi-starvation conditions. Arabidopsis seedlings (Col-0), germinated on agar medium with 500 μM Pi (Pi-sufficient medium) for 5 days, were transplanted to medium with 500 μM, or 10 μM Pi, and then collected over a 7-day time course of Pi-starvation treatment. The qRT-PCR analysis was performed with the same materials used in Figure 2M-N, to confirm that Pi-starvation treatment enhanced *PHT1;4* in shoots and roots of Arabidopsis seedlings. The Arabidopsis *Actin* gene was used as an internal control to normalize the qRT-PCR results. The data are presented as the mean ± SD (three biological replicates and three technical repeats).

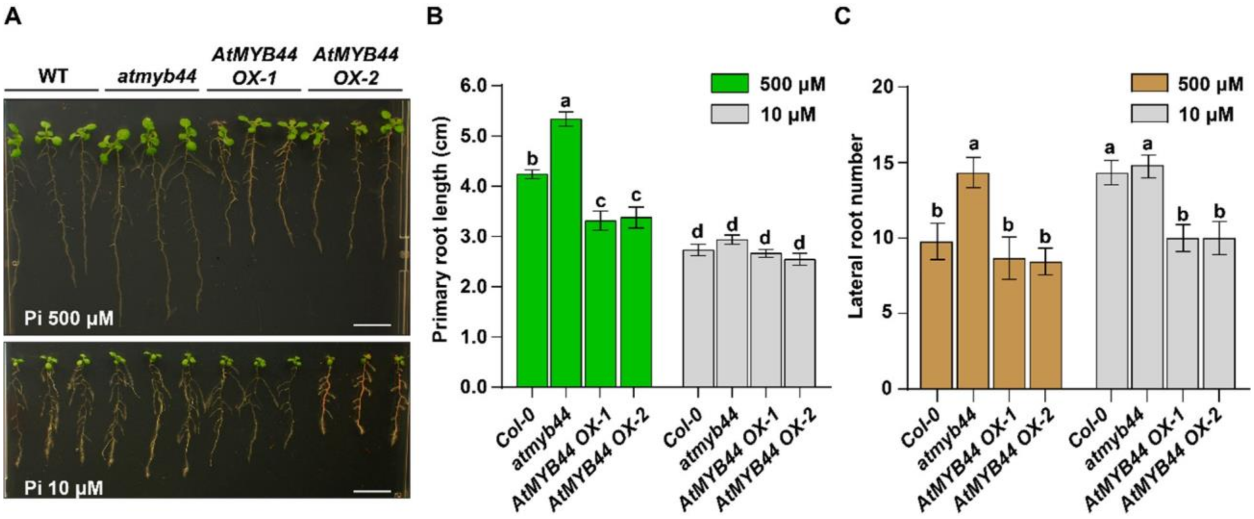

Figure S3. AtMYB44 is involved in primary and lateral root development.

(A) *atmyb44* displays enhanced primary root growth under Pi-sufficient (500 µM) conditions compared to WT and OX lines. WT, *atmyb44*, *AtMYB44 OX-1* and *AtMYB44 OX-2* were grown on Pi-sufficient (500 µM) and Pi-starvation (10 µM) media for 11 days after germination. Bar = 1 cm.

(B) Primary root length of WT, *atmyb44, AtMYB44 OX-1* and *AtMYB44 OX-2* seedlings, grown on the agar media with 500 µM, or 10 µM Pi, was measured 11-day after germination (DAG) (n = 12).

(C) *atmyb44* shows enhanced lateral root formation under Pi-sufficient (500 µM) conditions.

The different lowercase letters indicate significant differences in primary root length (B) and lateral root number (C), determined by the Tukey's test ($p<0.05$).

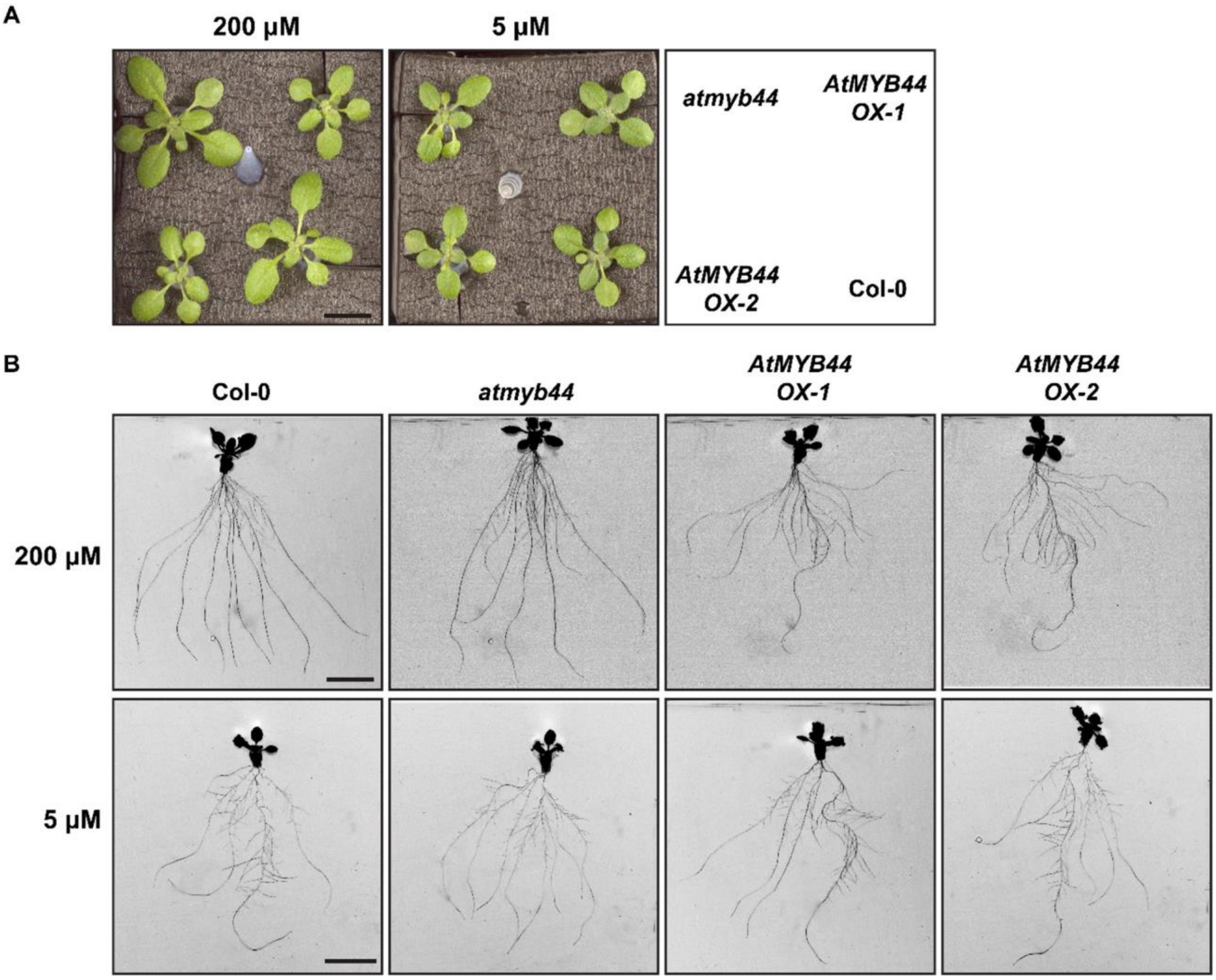

Figure S4. AtMYB44 serves as a potential negative regulator in shoot and root development.
Representative shoot (A) and root (B) images of WT, *atmyb44*, *AtMYB44* OX-1 and *AtMYB44* OX-2 plants grown in a hydroponic culture system with Pi-sufficient (200 μM, left panel in Figure S3A, upper panel in Figure S3B) and -starvation (5 μM, middle panel in Figure S3A, lower panel in Figure S3B) treatment. The right panel of Figure S3A indicates the position of WT, *atmyb44*, *AtMYB44* OX-1 and *AtMYB44* OX-2 plants in the hydroponic culture system. Bar = 1 cm.

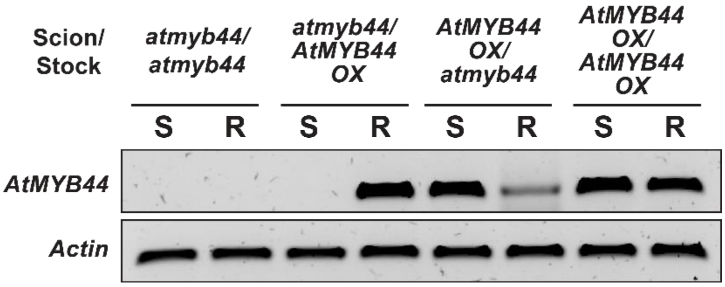

Figure S5. Mobility of *AtMYB44* mRNA.
AtMYB44 is graft-transmissible from the *AtMYB44 OX-1* scion into the *atmyb44* rootstock.

Table S1. List of PCR primers used in this study

Primer Name	Oligonucleotide Sequence 5-3'	Target Gene Name
055F_AtACT2	GAAATCACAGCACTTGCAC	AtActin2
055R_AtACT2	AGCCTTTGATCTTGAGAG	AtActin2
F_AtMYB44	TCTCCACCTGTTGTTACTGGGCTT	AtMYB44
R_AtMYB44	TTGACTCGTGGCTACGGTTTGACT	AtMYB44
060F_AtMYB70	GGCGACGATTGCACGGCTTC	AtMYB70
060R_AtMYB70	CCATCATACCCTCCATTACCACCG	AtMYB70
061F_AtMYB73	GCAGCGTCGAAGGGCAAAGT	AtMYB73
061R_AtMYB73	CCGATGGACTTCCGGGACTCA	AtMYB73
048F_AtMYB77	TGTCTTCGTCTTCGGAGGAT	AtMYB77
048R_AtMYB77	CCGCCATGTAACTCCTCACT	AtMYB77
086F_AtPHT1;4	TGACGTGGCCAAGACGCAAT	AtPHT1;4
086R_AtPHT1;4	GGCTTTTGTGCGCCTTCCAC	AtPHT1;4
088F_proAtMYB44	CACCAAGATGAAATAGTACTTG	AtMYB44 promoter
088R_proAtMYB44	TGATTTTGGAATGTTTTATCAAA	AtMYB44 promoter
Pht1-2-F	GCAACGAAGCCAAGGGTGTC	AtPHT1;2
Pht1-2-R	GAAAGCCCCACGGGTCTTCT	AtPHT1;2
MGD3-F	CCATCTTTCCCTCGCACTATTCT	AtMGD3
MGD3-R	ATCTCCAAGGGCTAGAGCTGTTT	AtMGD3
